# Glove Technique in Single-Port Access Laparoscopic Surgery: Results of an Initial Experience

**DOI:** 10.1155/2012/415430

**Published:** 2012-04-05

**Authors:** Lorenzo Livraghi, Mattia Berselli, Veronica Bianchi, Lorenzo Latham, Luca Farassino, Eugenio Cocozza

**Affiliations:** Dipartimento di Chirurgia, Azienda Ospedaliera, Ospedale di Circolo e Fondazione Macchi, 21100 Varese, Italy

## Abstract

*Introduction*. Single-incision laparoscopic surgery (SILS) is a virtually “scarless” technique. A retrospective analysis is performed to evaluate an initial experience of this surgical approach. *Materials and Methods*. From January 2010 to October 2011, SILS was considered as a minimally invasive approach to abdominal disease. The access was made by a standard wound protector and a size 6 glove. A series of little accesses were made on the tips of the glove-fingers to induce pneumoperitoneum and to create a working channel for the laparoscopic instruments. An analysis of costs of this technique was made too. *Results*. SILS was successfully completed with low cost in 34 patients: 20 appendectomy, 12 cholecystectomy, and 2 right colectomy were performed with a median operative time of 35, 45, and 67.5 minutes, respectively. In no patient any conversion to standard laparoscopy or to open surgery was needed. The postoperative course was uneventful in all patients. In right hemicolectomy, the oncological parameters were respected. *Conclusions*. In this paper the glove-port technique showed multiple advantages. The SILS is a feasible approach for some pathologies in selected patients. The glove-port is a simple, low-cost, reproducible, and sure method to perform SILS in a high-experienced laparoscopic surgical centre.

## 1. Introduction

Laparoscopic surgery is a well-established surgical technique for a variety of procedures. In recent years, multiple attempts to decrease parietal trauma and visible scars have been proposed. These efforts include the reduction of the diameter of the port size, the reduction in the number of the laparoscopic access [1–5], and the introduction of natural orifice transluminal endoscopic surgery (NOTES) [6–8] and of single incision laparoscopic surgery (SILS) [9–12]. SILS is a virtually “scarless” technique; the single port is hidden in the umbilicus. It is a rapidly evolving field: this approach is recently under investigation in some laparoscopic surgical centres to achieve less postoperative pain, less discomfort, and fewer surgical scares.

In a laparoscopic centre, a retrospective analysis is performed to evaluate an initial experience in laparoscopic surgery with the single-port technique and a periumbilical access; a detailed description of the SILS approach as a simple, safe, and cheap technique is done.

## 2. Patients and Methods

### 2.1. Patients

 In a surgical centre from January 2010 to October 2011 SILS was considered for minimally invasive approach for abdominal disease. All patients underwent surgery after obtaining an informed consent. A Patients selection was made before deciding the proper surgical approach. Exclusion criteria for minimally invasive approach were the same of traditional laparoscopic surgery.

Clinical or radiological signs of complicated appendix or gallbladder disease (masses and abscesses) and of voluminous neoplasms, the presence of liver cirrhosis, peritonitis, previous upper abdominal surgery, or severe obesity were exclusion criteria for SILS.

### 2.2. Single-Port Access Technique: Surgical Glove Port Construction

**Figure 1:**
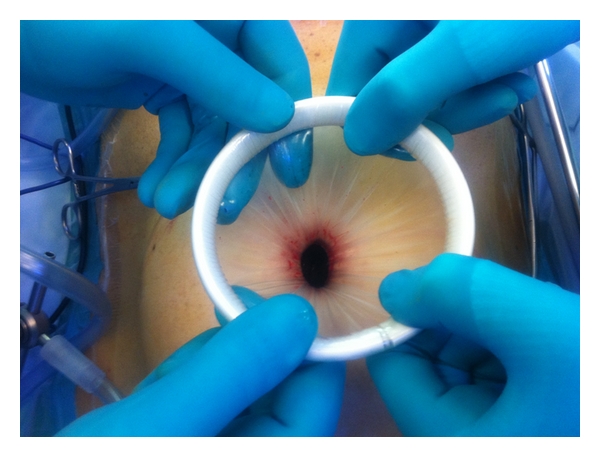
Placement of wound protector.

**Figure 2:**
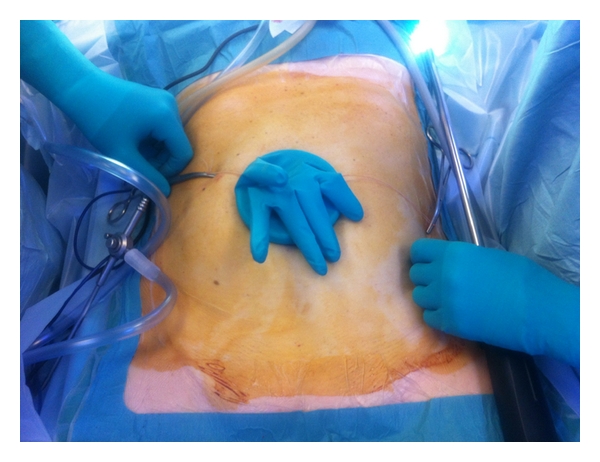
Placement of surgical glove.

**Figure 3:**
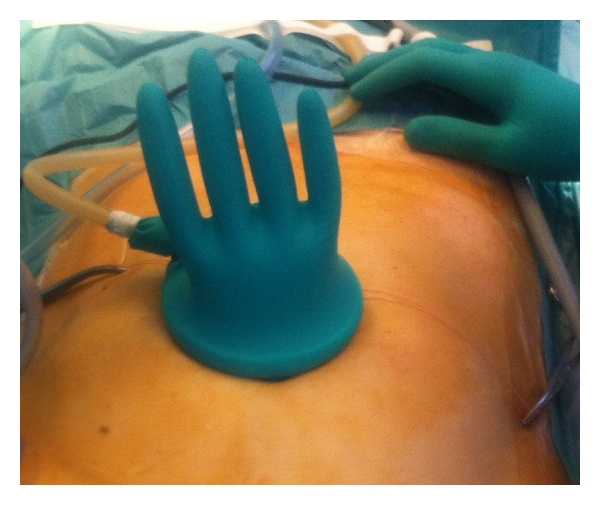
Induction of pneumoperitoneum.

**Figure 4:**
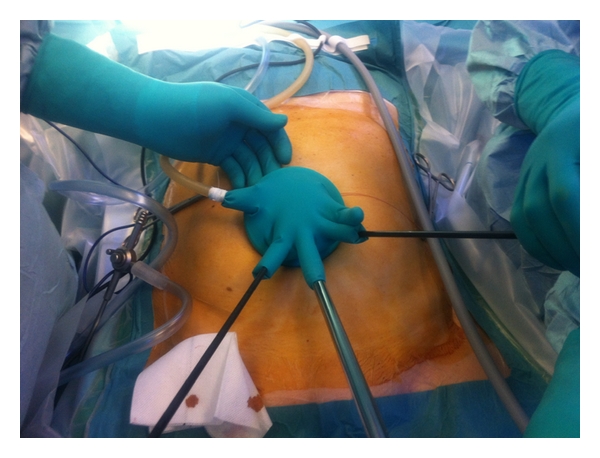
Placement of instruments.

An access device was made by a standard wound protector (a small size or extra small size ALEXIS wound retractor; Applied Medical, CA, USA) (Figure 1) and size 6, nonlatex sterile glove. The wound retractor was introduced through the small umbilical incision. The surgical glove was fixed to the outer ring of the wound retractor (Figure 2). A little access was made on the tip of one finger, and the CO2 pipe was connected to induce pneumoperitoneum (Figure 3). Other accesses were made on the others fingers to create a working channel for the laparoscopic instruments (Figure 4). Five- or three-millimeter traditional or curved laparoscopic instruments were used.

## 3. Results

SILS was successfully completed in 34 patients: 20 appendectomy was performed in female patients (median age 15, range 9–32 years), cholecystectomy in 12 patients (11 female and 1 male, median age 35, range 17–83 years), and right hemicolectomy in 2 female patients (55 and 64 years old).

In no patient conversion to standard laparoscopy or to open surgery was needed.

The median operative time for appendectomy, cholecistectomy and right hemicolectomy was 35, 45, and 67.5 minutes, respectively.

**Figure 5:**
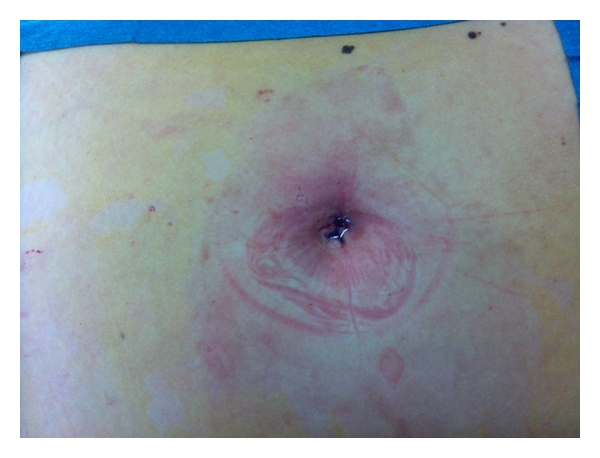
The umbilical scare at the end of a procedure.

Blood loss was minimal in all cases. No wound complication occurred; a picture of the scare at the end of a procedure is showed in the Figure 5.

The postoperative course was uneventful in all patients. The median postoperative in-hospital stay was 2 days for appendectomy and cholecistectomy and 6 days for right hemicolectomy.

The characteristics of patients and the perioperative results are resumed in Table 1.

An analytical analysis of postoperative pain was not performed; however, no patient needed any opiates drugs and no discharged was conditioned by sorrow.

In right hemicolectomy, the resection margins were oncologically correct and the number of regional limphonodes was adequate: in the surgical specimen of the first patient, 17 limphonodes were found with 2 micrometastases; in the second patient, 14 limphonodes were found without any sign of disease. An adequate preoperative staging was performed: thoracic and abdominal CT with contrast enhancement and colonoscopy excluded, respectively distant metastases and other cancer colonic localization.

An analysis of costs of this technique was made too. The prices of wound protector and of glove are respectively 50 and 0,51 euro (IVA 21% Excluded).

## 4. Discussion

A series of 34 patients underwent SILS with “Glove Technique” in a General Surgery Unit: postoperative complication rate was nil, oncological requires were respected in approaching to right colon neoplasms, and, furthermore, this technique is cheaper.

The procedures did not seem to take longer than expected for traditional laparoscopic approaches. Each intraoperative step was accomplished with confidence, similar to standard multiport laparoscopy. These results are in accordance with those reported in the literature: the use of the “glove-port” has been reported previously in general surgery [13–15] studies as in others specialities; in some papers it is moving from single-case descriptions to case series [16, 17].

In this paper the glove-port technique showed multiple advantages. It is easy to use and can be simply accommodated to the abdominal wall even in overweight patients. The glove-port allows simultaneous passage of several laparoscopic instruments through one small incision, and this fact can have several merits: the effect of the two rings of the wound retractor can prevent subcutaneous emphysema, port-site infection and bleeding. The umbilical incision is minimized; this advantage can decrease the postoperative pain and the rate of surgical site hernia development.

Many devices have three or four ports, whereas the glove-port allows to use simultaneously up to five instruments without any size limit. A wide axis of movements is possible with the glove-port technique: the instruments inside the abdomen can be used apart, easily crossed or rotated as required in any situation.

The cost of technique is very low, and this can be an advantage compared to the prices of some commercial dedicated devices.

The glove is not certified for this use, and the single-port access needs to be considered as advanced operative technique. The use of surgical glove obviates issues of devices cost but of course not operative skills. Intra-abdominal smoke that may slow the procedure somewhat is another problem because there is no separate venting channel.

A significant coordination between the surgeon and the camera holder is needed. The surgeon also has to be adapted to counterintuitive movements due to frequent crossing of the instrument shafts at the point of entry into the abdominal cavity.

Finally, if the lack of a fixed axis for instruments can be an advantage for movements as above discussed, it can cause in some conditions a further difficulty for the surgeon: the glove cannot always give just the same stability of a traditional trocar or single-incision device.

## 5. Conclusions

The SILS is a feasible approach for some pathologies in selected patients. The glove-port is a simple, reproducible and sure method to perform SILS in a high-experienced laparoscopic surgical centre. Further studies are necessary to demonstrate the advantages in terms of pain control, patient satisfaction, and surgical-related morbidity.

## Figures and Tables

**Table 1:** Patients and perioperative results.

	Cholecistectomy	Appendectomy	Right colectomy
Number of patients	12	20	2
Median age	26	15	59,5
Conversion to standard laparoscopy or to open	NO	NO	NO
Median operative time	45	35	67,5
Postoperative complications	NONE	NONE	NONE
Median postoperative in-hospital stay	2	2	6
